# Supplementary material for: Computational investigation of hysteresis and phase equilibria of n-alkanes in a metal-organic framework with both micropores and mesopores
Source: Commun Chem. 2023 May 8;6:90. doi: 10.1038/s42004-023-00889-3 (PMC10167368; doi:10.1038/s42004-023-00889-3)
Supplement: Supplementary file 2 — Supplemental information [file 42004_2023_889_MOESM2_ESM.pdf]

Supporting Information

Computational investigation of hysteresis and phase equilibria of n-alkanes in a metal-organic framework with both micropores and mesopores

Zhao Li, Jake Turner, and Randall Q. Snurr*

*Corresponding Author: snurr@northwestern.edu

Department of Chemical and Biological Engineering, Northwestern University, 2145 Sheridan Road, Evanston, IL 60208, USA

Table S1. Lennard-Jones parameters of n-alkane and framework pseudo-atoms.

Atom Type	ϵ/k_B (K)	σ (Å)
H	22.14	2.57
N	34.72	3.26
C	52.83	3.43
Zn	62.39	2.46
CH_4_sp3	148.0	3.73
CH_3_sp3	98.0	3.75
CH_2_sp3	46.0	3.95
CH_3_sp3 (TraPPE_UA2)	134.5	3.52
CH_4_sp3 (IRMOF-1 adsorption, shifted)	158.5	3.72
Zn (IRMOF-1 adsorption, shifted)	0.42	2.7
O1 (IRMOF-1 adsorption, shifted)	700.0	2.98
O2 (IRMOF-1 adsorption, shifted)	70.5	3.11
C1 (IRMOF-1 adsorption, shifted)	47.0	3.74
C2 (IRMOF-1 adsorption, shifted)	47.86	3.47
C3 (IRMOF-1 adsorption, shifted)	47.86	3.47
H1 (IRMOF-1 adsorption, shifted)	7.65	2.85

For the bonded terms of IRMOF-1, the interactions are composed of bond stretching, angle bending, torsion, and improper torsion interactions.

For the bond stretching, a harmonic potential was used:

$$\mathcal{V} = \frac{1}{2}k(r - r_0)^2 \tag{S1}$$

where $\mathcal{V}$ is the potential energy of the bond, k is the spring constant, r is the distance between the two atoms, and r_0 is the reference length of the bond.

Table S2. Parameters for the harmonic bonds in IRMOF-1. The parameters are taken from Dubbeldam et al.[1]

Bond Type	$k/\mathrm{k_B}$ (K/Å^2)	r_0 (Å)
C3-H1	366001.1	0.95
C3-C3	483413.9	1.36
C2-C3	483413.9	1.36
C1-C2	353750.9	1.42
O2-C1	543840.6	1.25

The bending interactions also used a harmonic potential:

$$\mathcal{V} = \frac{1}{2}k(r - r_0)^2 \tag{S2}$$

where $\mathcal{V}$ is the potential energy of the bend, k is the spring constant, θ is the angle, and θ_0 is the reference angle.

Table S3. Parameters for the harmonic bends in IRMOF-1. The parameters are taken from Dubbeldam et al.[1]

Bend Type	k/k_B (K/rad^2)	θ_0 (degree)
C1-C2-C3	34926.55	120
C2-C3-H1	37263.16	120
C3-C3-H1	37263.16	120
C3-C2-C3	90640.11	120
C3-C3-C2	90640.11	120
O2-C1-O2	135960.2	130
O2-C1-C2	54882.48	115

The torsion and improper torsion interactions are defined as:

$$\mathcal{V} = k[1 + \cos(m\phi - \phi_0)] \tag{S3}$$

Table S4. Parameters for the torsions in IRMOF-1. The parameters are taken from Dubbeldam et al.[1]

Torsion Type	k/k_B (K)	m	ϕ_0 (degree)
O2-C1-C2-C3	1258.89	2	180
C1-C2-C3-H1	1510.67	2	180
C1-C2-C3-C3	1510.67	2	180
H1-C3-C3-H1	1510.67	2	180
C2-C3-C3-H1	1510.67	2	180
C2-C3-C3-C2	1510.67	2	180
H1-C3-C2-C3	1510.67	2	180
C3-C2-C3-C3	1510.67	2	180

Table S5. Parameters for the impropers in IRMOF-1. The parameters are taken from Dubbeldam et al.[1]

Improper Type	k/k_B (K)	m	ϕ_0 (degree)
C2-C1-O2-O2	5035.56	2	180
C3-C2-C3-C1	5035.56	2	180
C2-C3-C3-H1	186.32	2	180

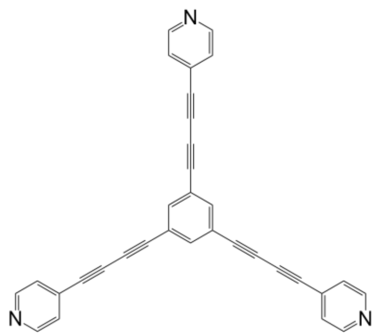

Figure S1. Linker in MOF # 667 in the ToBaCCo 1.0 database. MOF # 667 has a single zinc atom as the metal node. The SMILES string for this linker molecule is C1(C#CC#CC2=CC(C#CC#CC3=CC=NC=C3)=CC(C#CC#CC4=CC=NC=C4)=C2)=CC=NC=C1.

Table S6. Textural properties of MOF #667 calculated via Zeo++.

MOF	Largest Cavity Diameter (Å)	Pore Limiting Diameter (Å)	Volumetric Surface Area (m^2/cm^3)	Gravimetric Surface Area (m^2/g)	Void Fraction
# 667	27.8	18.6	1001	8595	0.95

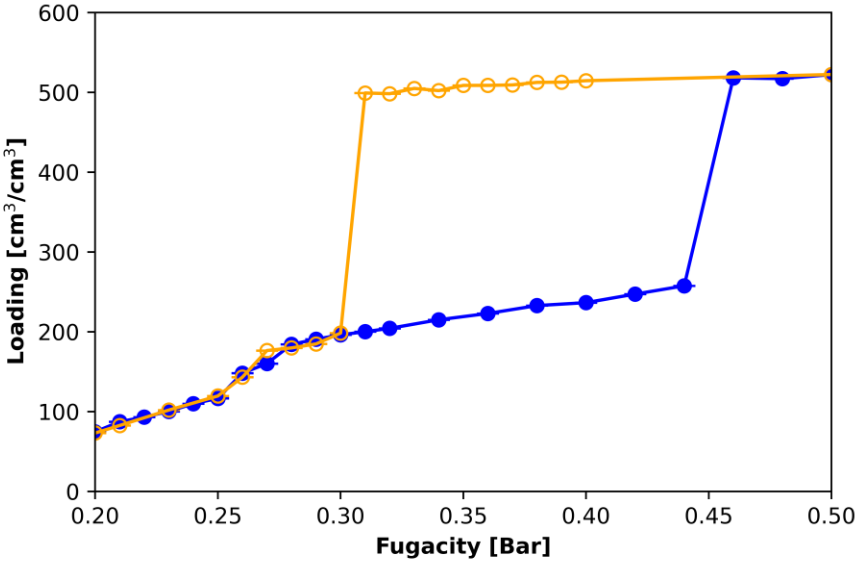

Figure S2. Adsorption and desorption isotherms for methane at 112 K (Tr = 0.587) between 0.2 bar and 0.5 bar. There is a very small hysteresis loop for the adsorption/desorption in the micropore.

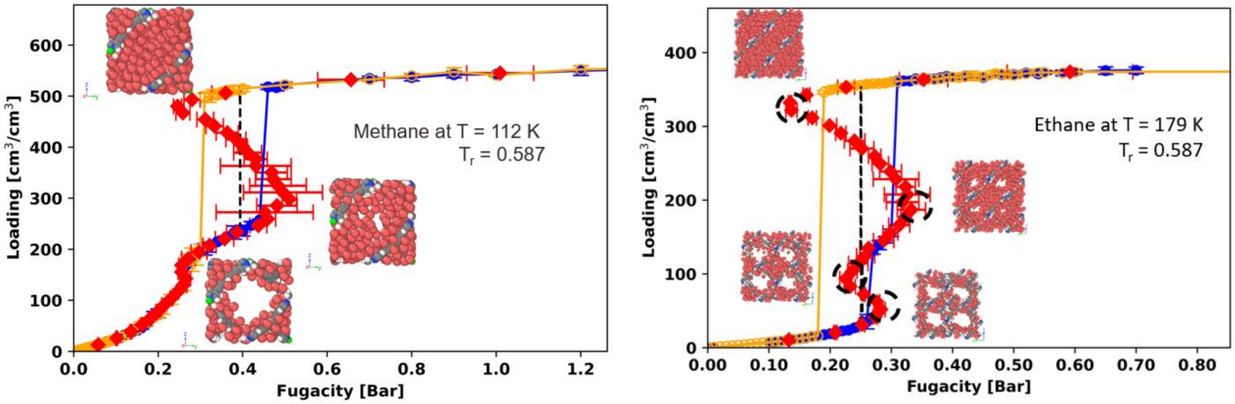

Figure S3. Isotherms and snapshots of methane in MOF #667 at 112 K (left) and ethane in the same MOF at 179 K (right). The blue and yellow points are the adsorption and desorption isotherms, respectively, from GCMC simulations, and the red points are the canonical isotherms. The error bars show twice the standard deviation in the predictions from the GCMC and canonical simulations. Black lines are the binodal transition calculated from Maxwell's construction of equal areas. Simulations were performed using 1 unit cell of the MOF. The snapshots correspond to the filling of the micropore, the low-density spinodal and the high-density spinodal of the mesopore. We used four unit cells for the

ethane snapshots to clearly show the difference between the low-density and high-density spinodal for the micropore.

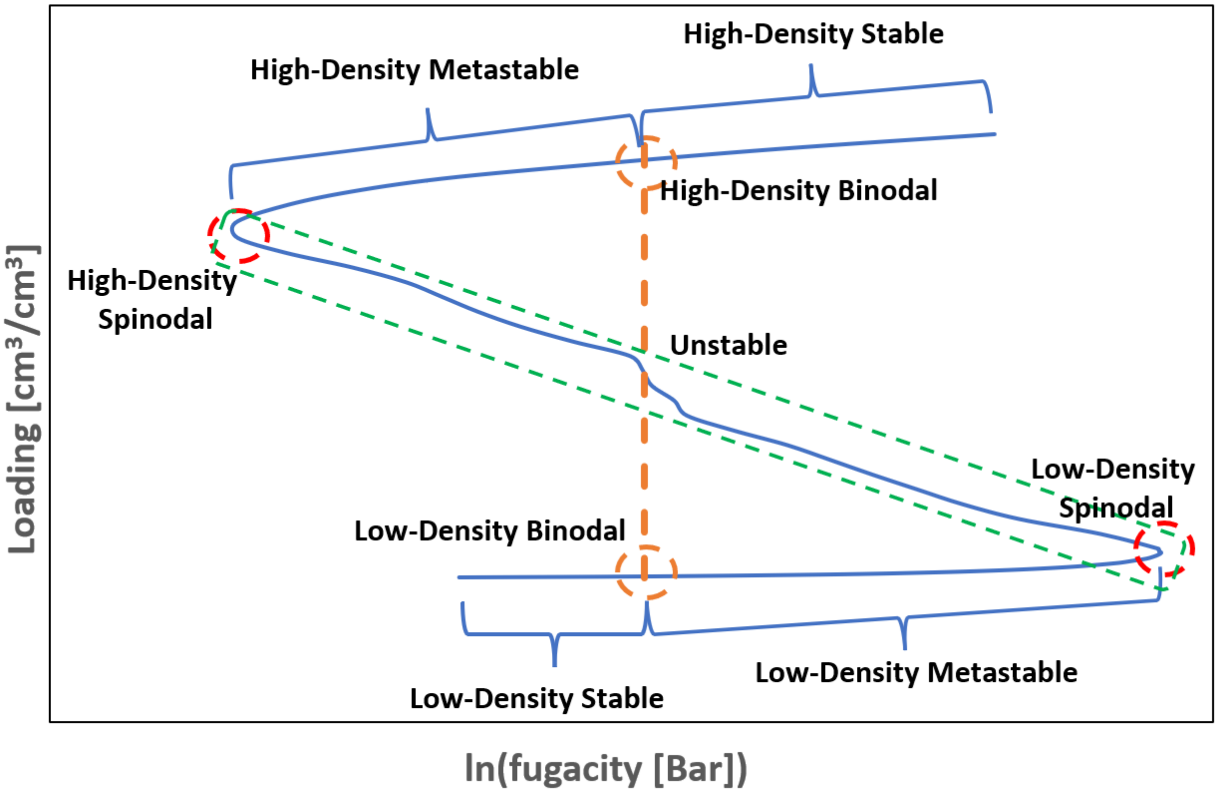

Figure S4. Schematic of different regions and terminology of the van der Waals loop.

Table S7. Experimental and TraPPE critical temperatures for methane, ethane, and propane. Note that we used the TraPPE-UA model[3] for methane and propane, and for ethane we used the TraPPE-UA2 model[5], which tuned the parameters for pseudo-atoms specifically for ethane.

Molecule	Experimental T_c [K]	TraPPE T_c [K]
Methane	190.6[2]	191.4[3]
Ethane	305.3[4]	308.6[5]
Propane	369.8[6]	368.2[3]

To obtain the bulk-phase binodal fugacities (f_0) for methane and ethane, similar to the method of obtaining binodal fugacities of adsorbates in MOFs, we performed Widom insertions in an empty simulation box at different numbers of methane or ethane molecules. The van der Waals loops of methane and ethane at different temperatures were obtained, and the f_0 values were calculated by Maxwell's construction.

Table S8. Fugacities of the low-density and high-density spinodals for each equilibrium (i.e., for an individual pore or overall) for the canonical isotherms reported in Figure 2 for methane and ethane at T_r = 0.587 and 0.701. The spinodal span column tells the range of the unstable region bounded by the spinodals, and it is the difference between the fugacities of the low-density and the high-density spinodals. The fugacities are divided by f_0 for methane and ethane at these two temperatures (reported in the last column).

Molecule-Reduced Temperature-Temperature-Pore	$\frac{f_{low-density\ spinodal}}{f_0}$	$\frac{f_{high-density\ spinodal}}{f_0}$	$\frac{f_{spinodal-span}}{f_0}$	f_0 (No tail-correction) [bar]
C1-0.587-112 K-Micropore	0.210	0.202	0.008	1.263
C1-0.587-112 K-Mesopore	0.404	0.195	0.209	1.263
C1-0.701-134 K-Mesopore	0.463	0.373	0.089	4.923
C2-0.587-179 K-Micropore	0.365	0.309	0.056	0.854
C2-0.587-179 K-Mesopore	0.410	0.197	0.213	0.854
C2-0.587-179 K-Overall	0.410	0.197	0.213	0.854
C2-0.701-214 K-Mesopore	0.460	0.382	0.078	4.072

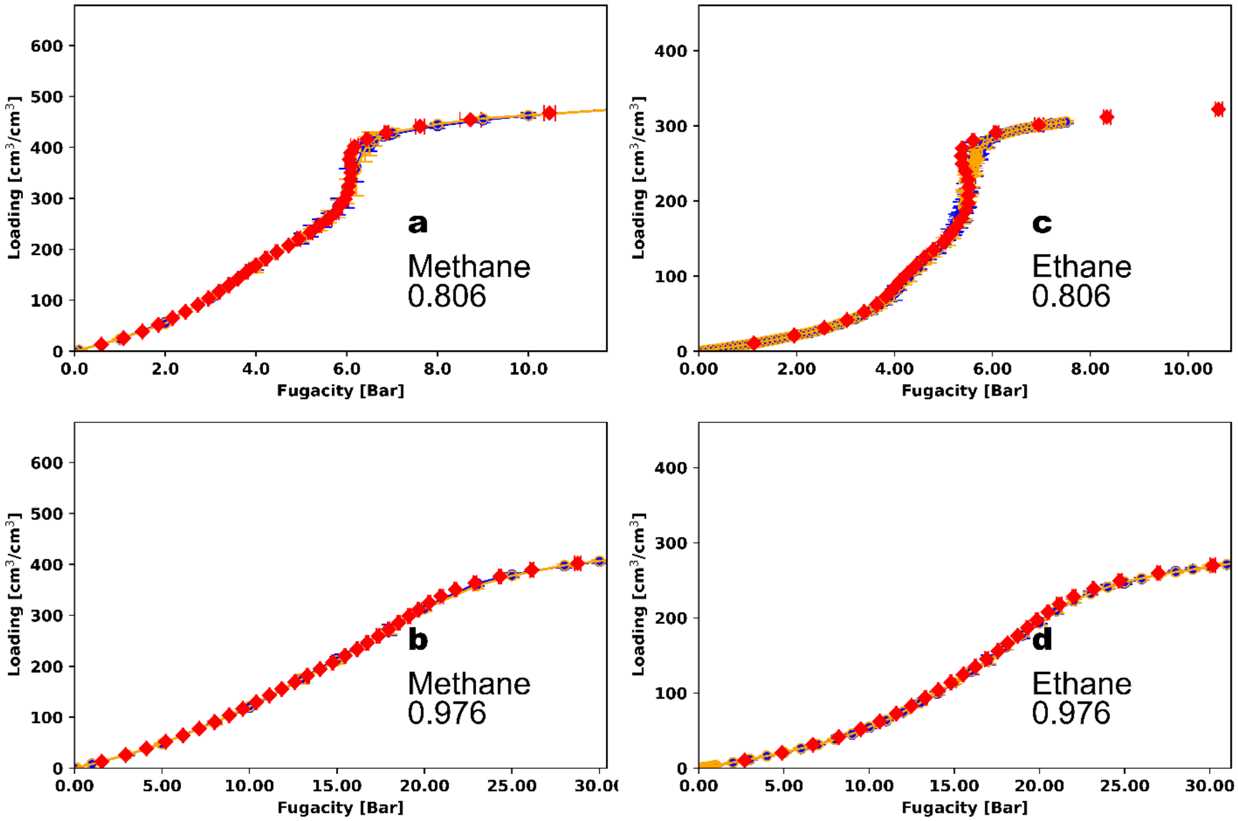

Figure S5. Methane (a and b) and ethane (c and d) isotherms in MOF #667 in the ToBaCCo 1.0 database at different reduced temperatures relative to their experimental critical temperatures (0.806 and 0.976). The red dots are the canonical isotherms, the blue and yellow curves are the adsorption and desorption isotherms, respectively, from GCMC simulations. The error bars show twice the standard deviation in the predictions from the GCMC and canonical simulations. The simulations used one unit cell.

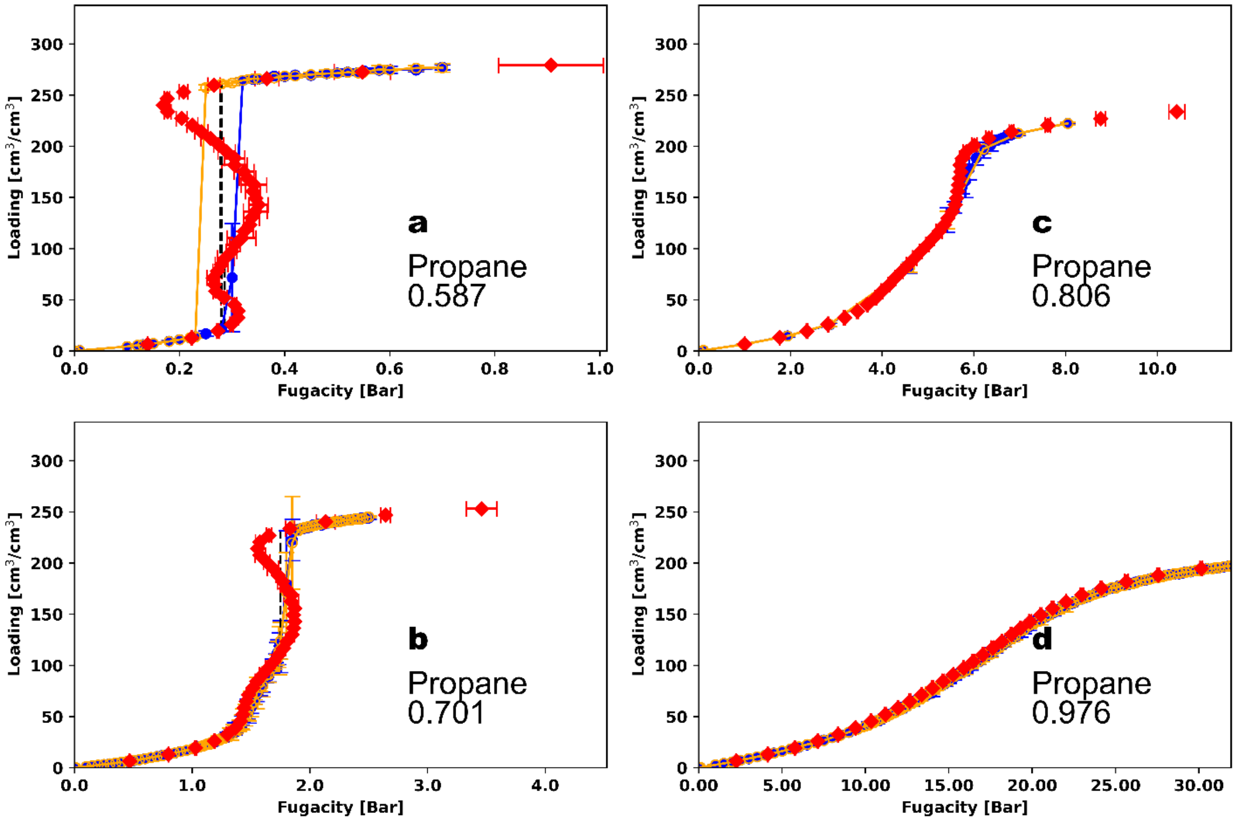

Figure S6. Propane isotherms in MOF #667 in the ToBaCCo 1.0 database at different reduced temperatures relative to their experimental critical temperatures (0.587, 0.701, 0.806, and 0.976). The red dots are the canonical isotherms, and the blue and yellow curves are the adsorption and desorption isotherms, respectively, from GCMC simulations. The error bars show twice the standard deviation in the predictions from the GCMC and canonical simulations. The simulations used one unit cell.

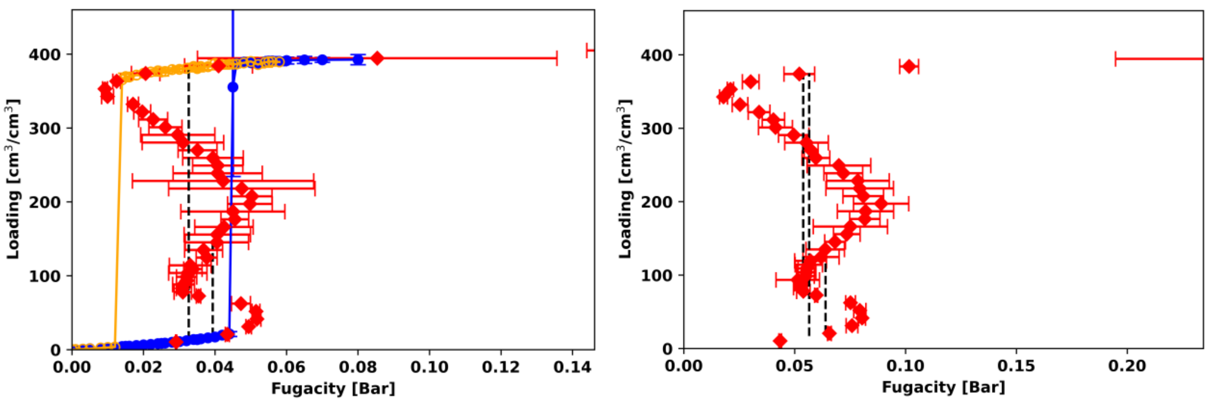

Figure S7. Ethane isotherms at T = 152 K (Tr = 0.5, left) and 159 K (Tr = 0.52, right). The red diamonds show the canonical isotherms, and the dashed vertical lines show the binodals for different pores and for the overall structure. For 152 K (T_r = 0.5), there is no binodal transition for the mesopore. The error bars show twice the standard deviation in the predictions from the GCMC and canonical simulations.

Enthalpy of Adsorption around the Step Change

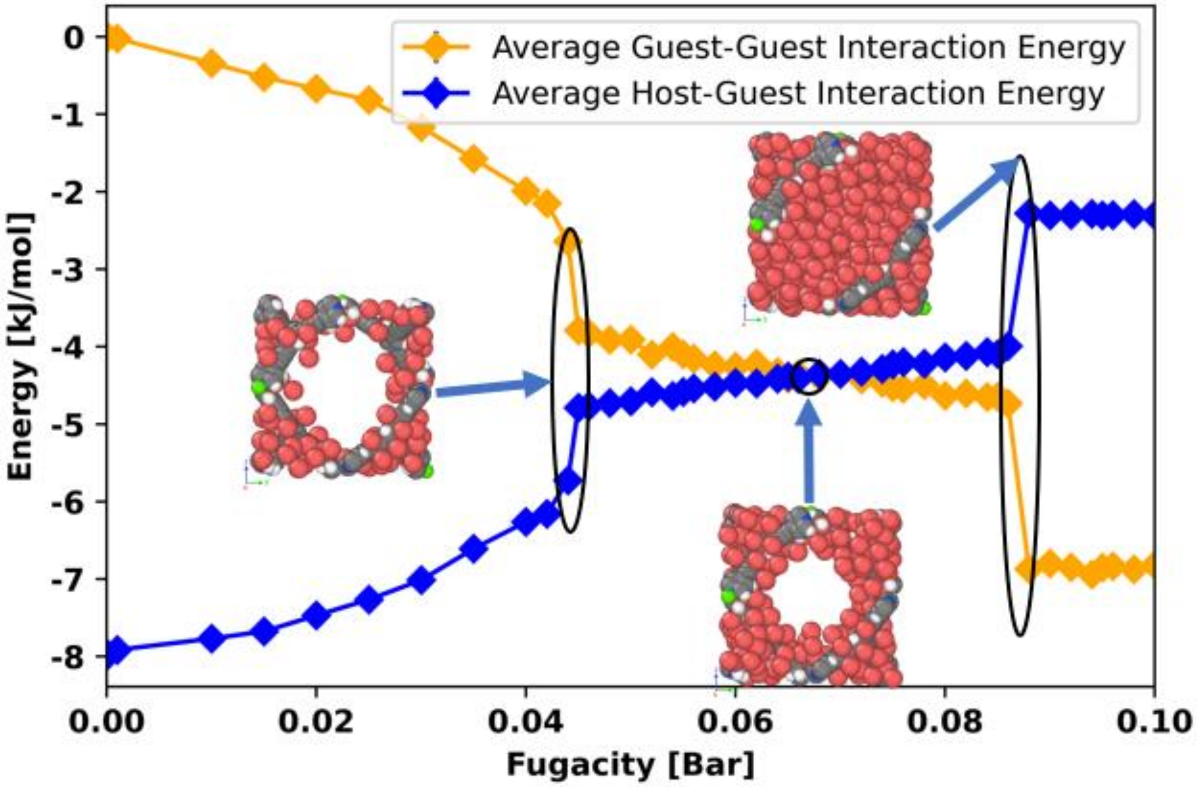

Figure S8. Average host-guest (blue) and guest-guest (orange) interaction energies from GCMC simulation of methane in MOF #667 at 95 K (T_r = 0.5) as a function of fugacity. The black ovals indicate the regions where the snapshots were taken from.

System Size Effects

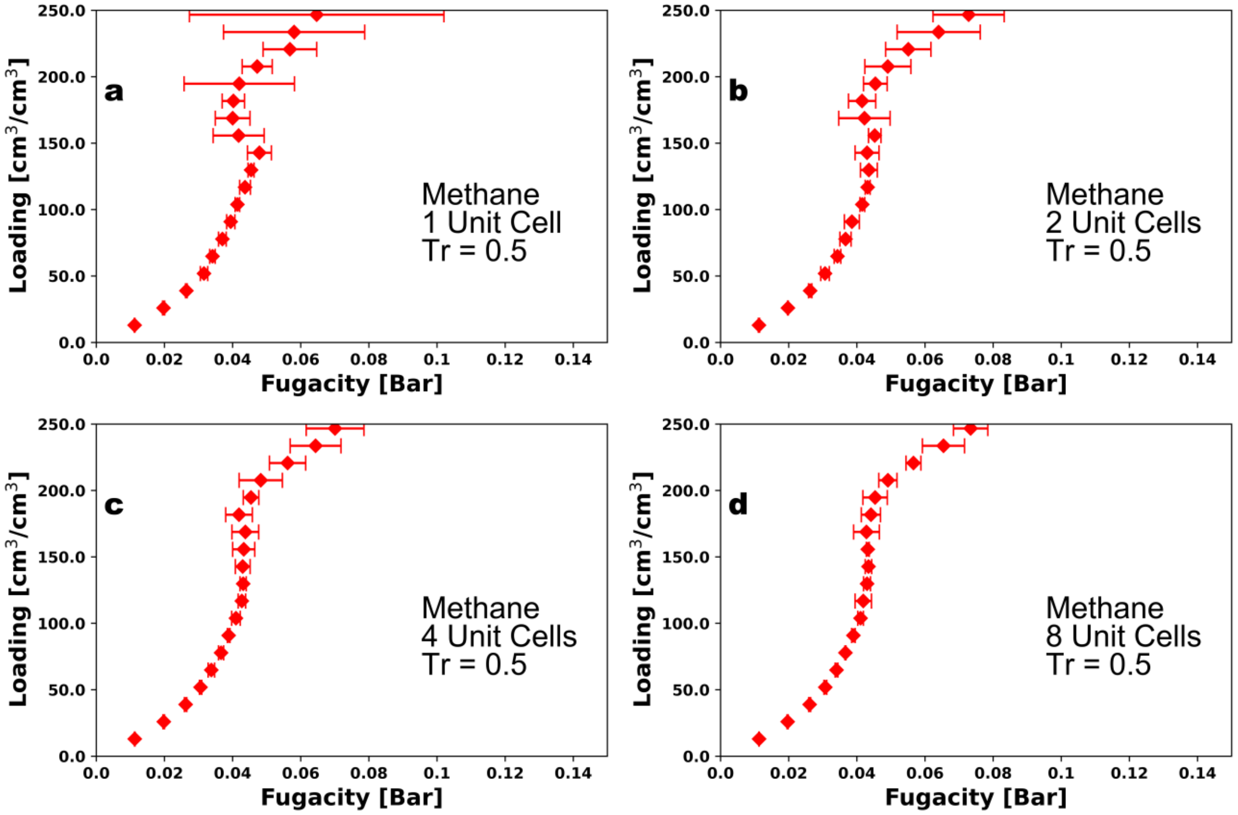

Figure S9. canonical isotherms for methane in (a) 1, (b) 2, (c) 4, and (d) 8 unit cells of MOF #667 at 95 K. These graphs zoom into the micropore region of MOF #667 to show how system size affects the van der Waals loop for the micropore. The error bars show twice the standard deviation in the predictions from the canonical simulations.

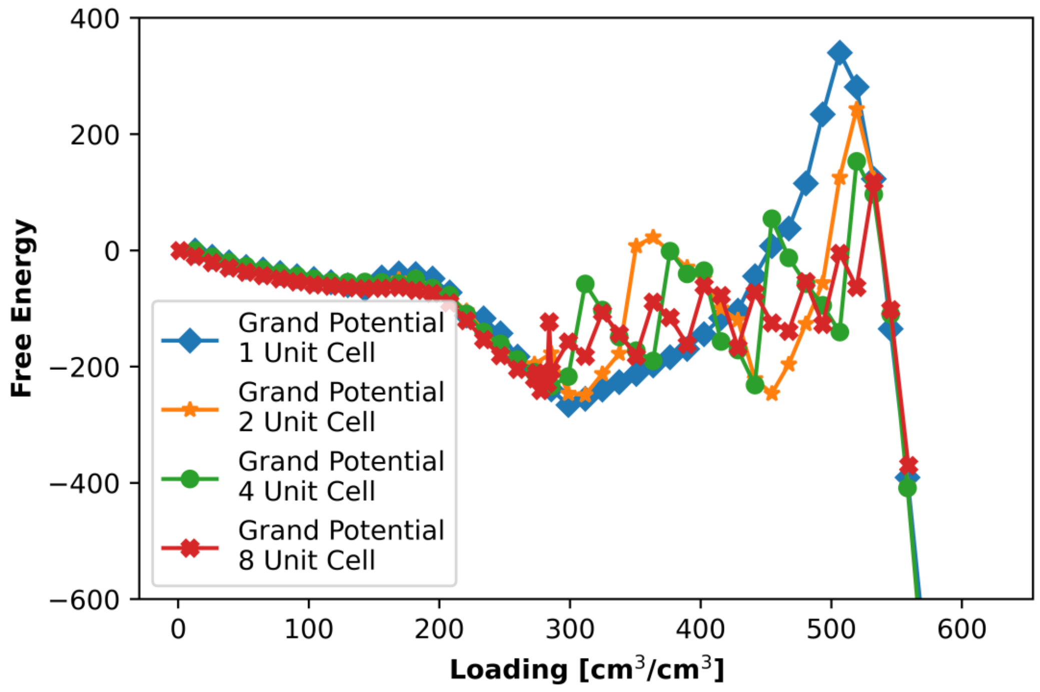

Figure S10. Grand potential versus loading for methane at 95 K in MOF #667 for 1 (blue diamonds), 2 (orange stars), 4 (green circles), and 8 (red crosses) unit cells.

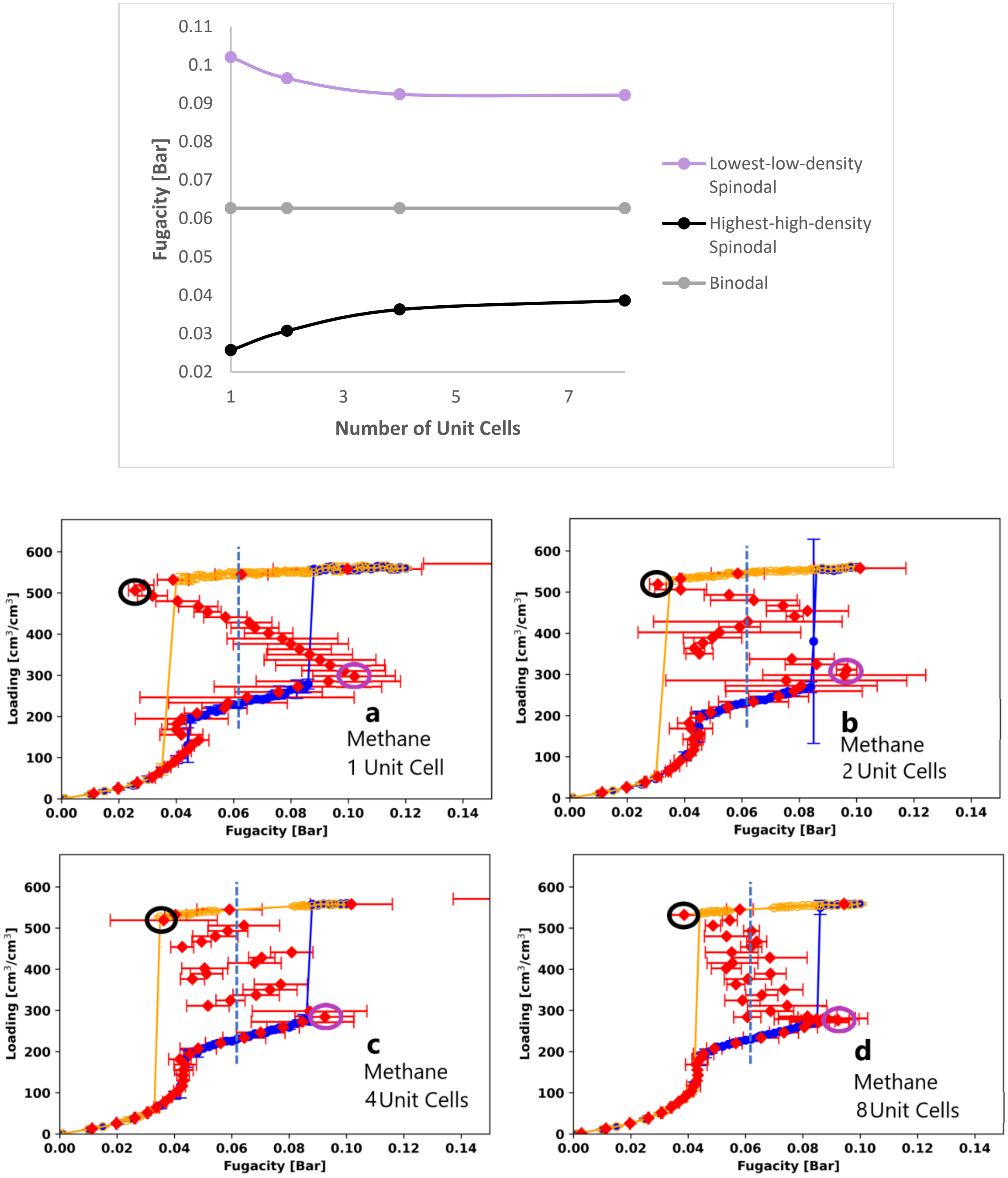

Figure S11. Fugacities of the lowest low-density (most bottom right red diamond in Figure 6) and the highest high-density (most top left red diamond in Figure 6) spinodals for the mesopore as a function of the number of unit cells used for methane in MOF #667 at 95 K. The error bars show twice the standard

deviation in the predictions from the GCMC and canonical simulations. As a reference, we added the binodal to the graph using the gray line. To show the points on the isotherms, we copied Figure 6 here and highlighted the spinodals using the same colors as in the top graph.

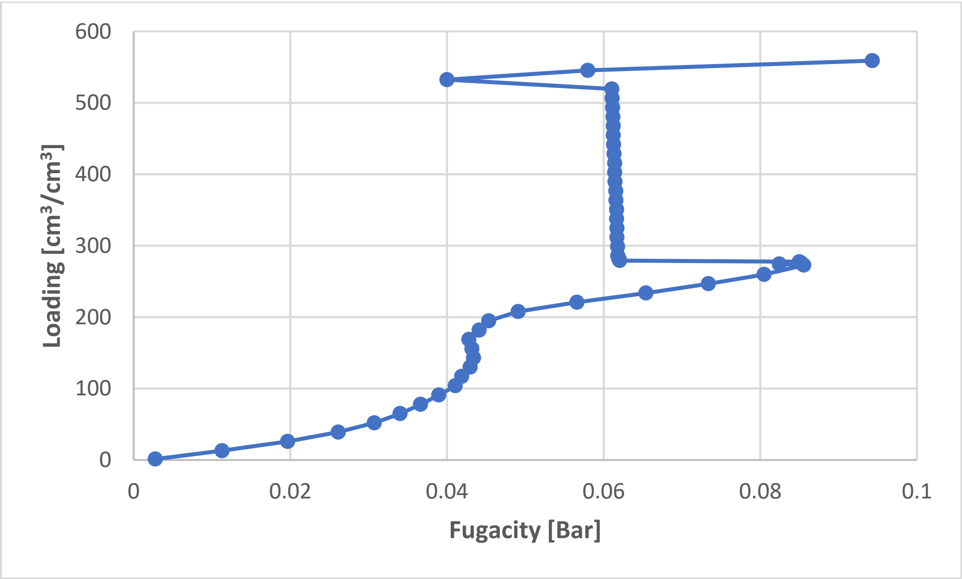

Figure S12. Schematic of canonical isotherm for methane in MOF #667 at 95 K extrapolated to infinite number of unit cells.

Effect of Tail Corrections

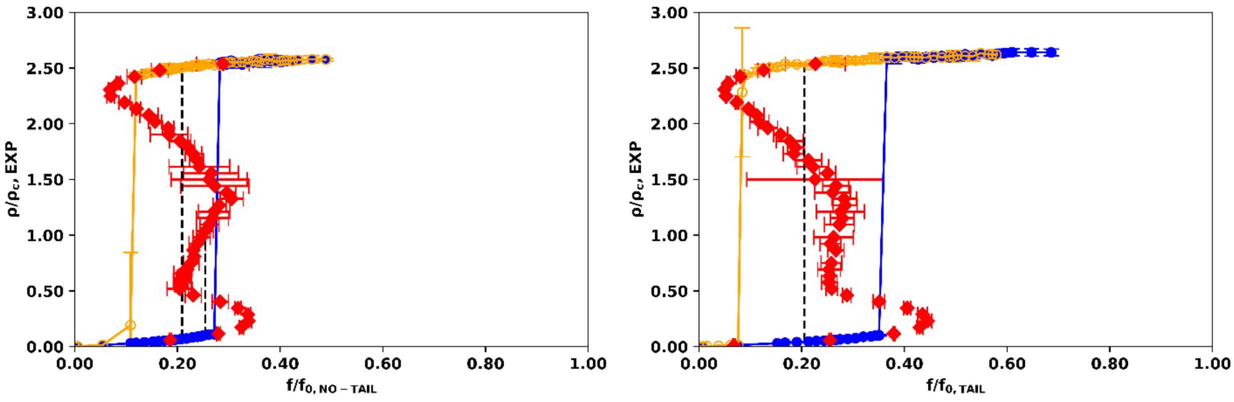

Figure S13. Comparison between the GCMC and canonical isotherms for propane in MOF #667 at 185 K (T_r = 0.5) without (left) and with (right) tail corrections. The error bars show twice the standard deviation in the predictions from the GCMC and canonical simulations. The x-axes are normalized by the bulk-phase binodal fugacities (f_0) for propane at 185 K (T_r = 0.5) with or without tail corrections. The y-axes are normalized by the experimental critical density of propane. The non-normalized isotherms are shown in Figure 10 in the main text.

Table S9. Comparison between the canonical isotherms for propane in MOF #667 at 185 K without and with tail corrections (Figure 10). We also included the bulk phase propane vapor fugacity (f_0) calculated with/without tail corrections at this temperature. Subscripts a, b, c, and d represent the spinodals in the isotherms from Figure 10.

	$f_{overall}$ (bar)	$\frac{f_{overall}}{f_0}$	f_a (bar)	f_b (bar)	f_c (bar)	f_d (bar)	$\frac{f_a}{f_0}$	$\frac{f_b}{f_0}$	$\frac{f_c}{f_0}$	$\frac{f_d}{f_0}$	f_0 (bar)
No Tail	0.039	0.212	0.013	0.056	0.038	0.062	0.070	0.306	0.204	0.338	0.184
Tail	0.027	0.205	0.006	0.037	0.033	0.059	0.0494	0.285	0.258	0.445	0.131

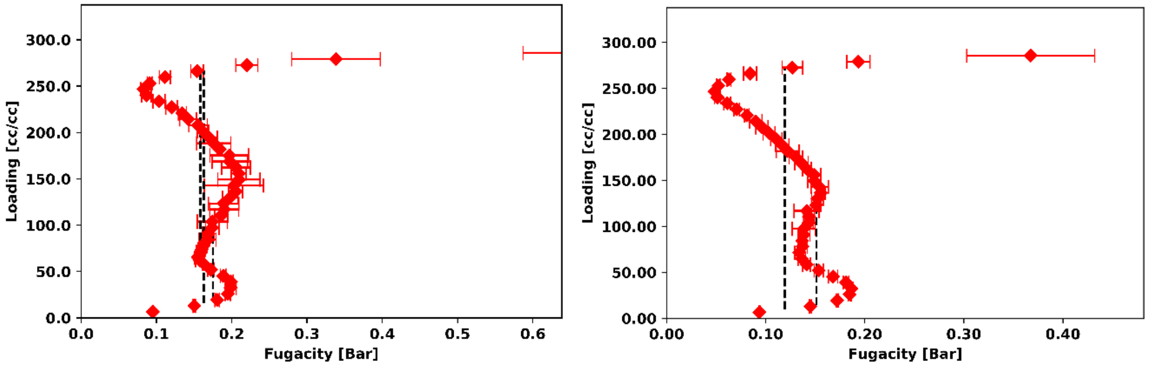

Figure S14. Propane canonical isotherms simulated at 207 K (T_r = 0.587) with no tail corrections (left) and with tail corrections (right). The error bars show twice the standard deviation in the predictions from the canonical simulation.

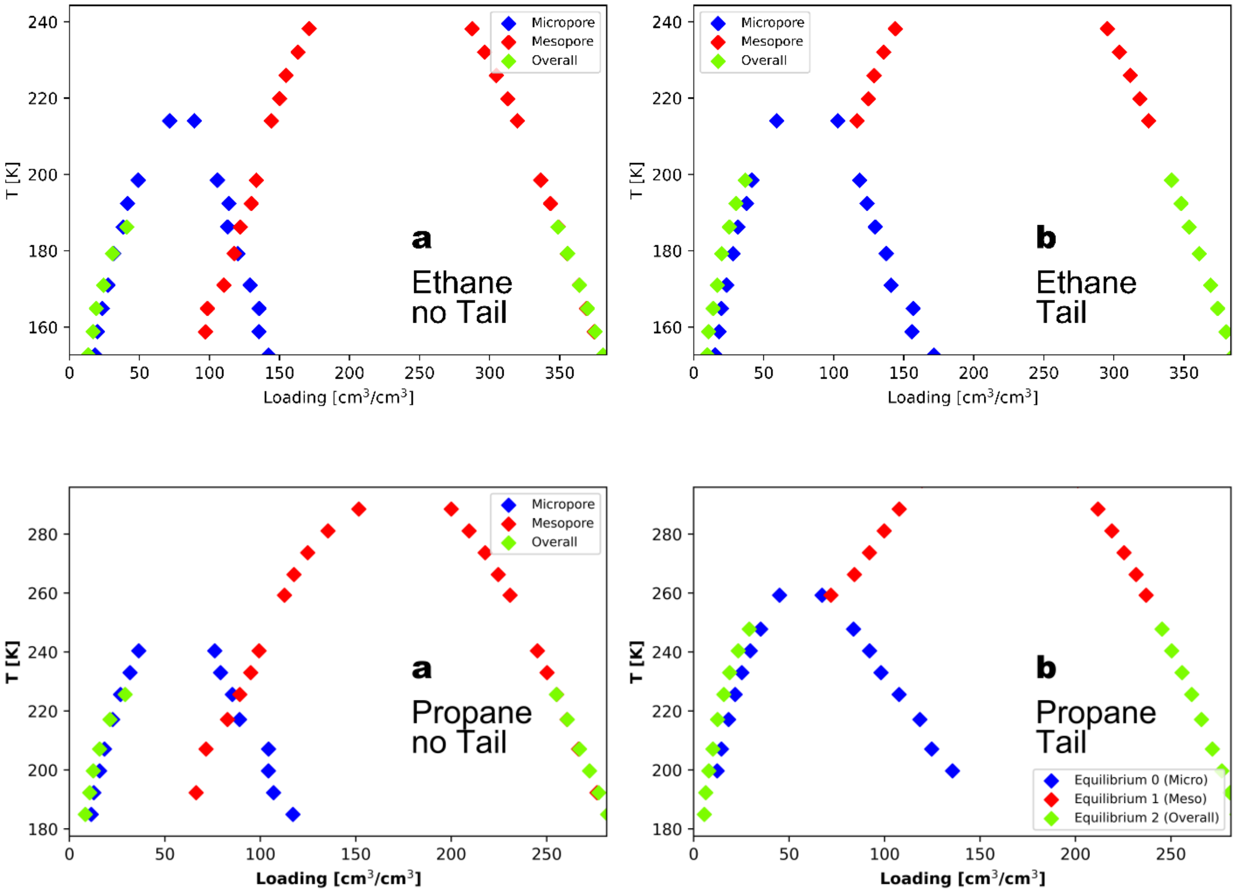

Figure S15. Phase equilibrium for ethane (first row) and propane (second row) in MOF #667 (1 unit cell) without (left) and with (right) guest-guest tail corrections.

In Figure S15, the high-density/low-density equilibrium for the micropore is shown in blue. The high-density branch is shifted to higher densities (loadings) when tail corrections are used, while the low-density branch is unchanged. The high-density/low-density binodal for the mesopore disappears for lower temperatures for the figure on the right. This can be observed for ethane and propane in MOF #667. As an example, a canonical isotherm of ethane at 159 K (T_r = 0.52) without and with tail corrections is shown in Figure S16. We can see that at 159 K, there is a clear difference when tail correction is used, and the mesopore binodal transition disappears. The cross-over between the low-density branch for the mesopore and high-density branch for micropore is not observable for the figure on the right (with guest-guest tail correction).

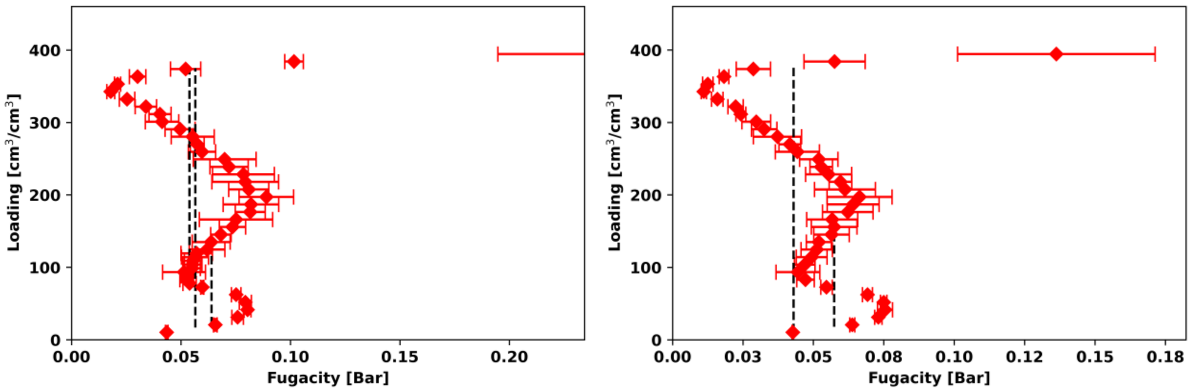

Figure S16. Ethane in MOF #667 at 159 K (T_r = 0.52) without (left) and with (right) tail corrections. The red diamonds represent canonical isotherms while the vertical dashed lines are the binodal transitions for the pores and the overall structure of MOF #667. The error bars show twice the standard deviation in the predictions from the canonical simulations.

We also observe in Figure S15 that if tail corrections are added, the overall equilibrium (green) cannot exist together with the mesopore equilibrium (red). The overall equilibrium merges with the mesopore equilibrium above the critical temperature for the micropore. For the canonical isotherms with tail

corrections, there are two equilibria, but there can be three if no tail corrections are used. See, for example, Figure S14 and Figure S16.

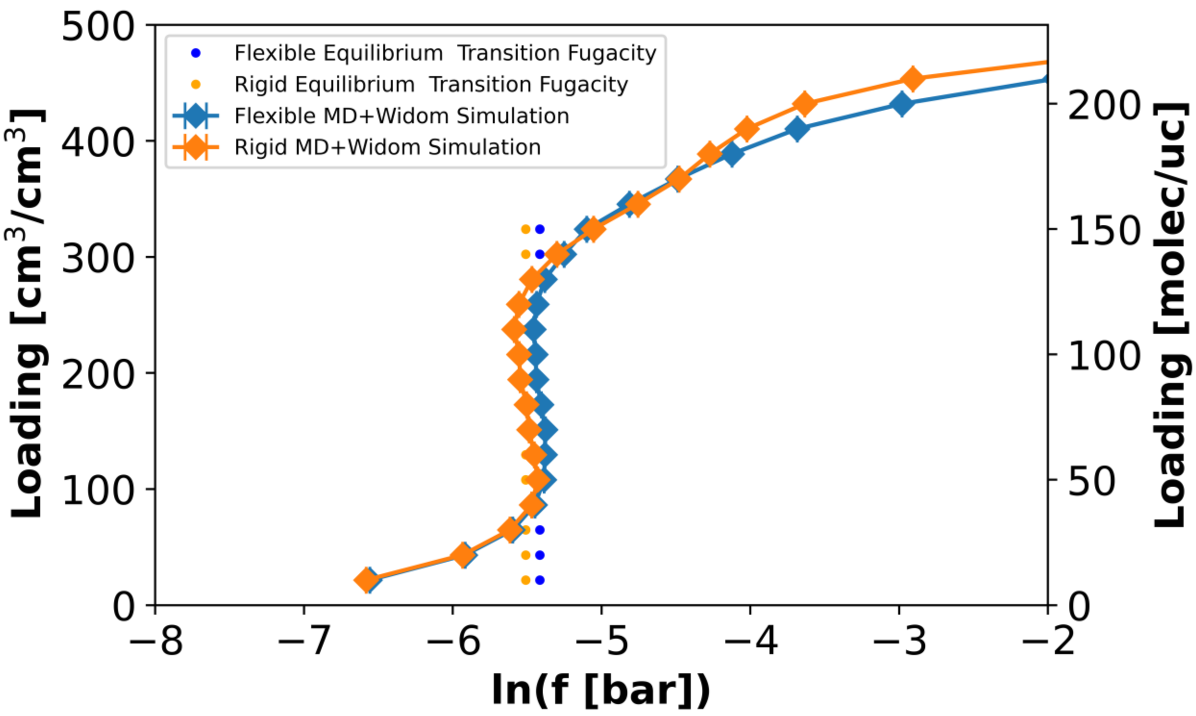

Figure S17. MD+Widom canonical isotherms for methane at 112 K (T_r = 0.587) in both rigid (orange) and flexible (blue) structure models of IRMOF-1. The dotted lines are the corresponding binodal transitions for the two framework models.

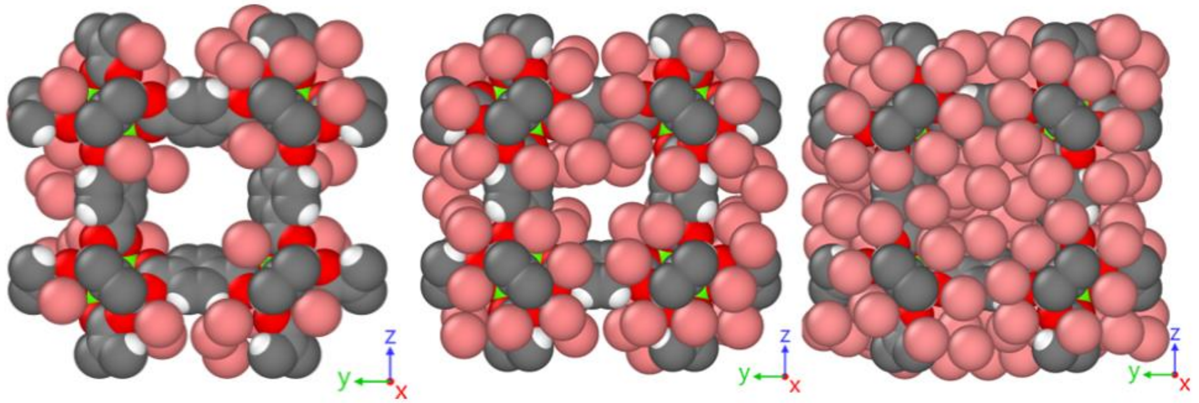

Figure S18. Snapshots from MD + Widom simulations for methane in rigid framework of IRMOF-1 at 112 K at loadings equal to 108 cm^3/cm^3 (50 molec/uc), 237 cm^3/cm^3 (110 molec/uc), and 432 cm^3/cm^3 (200 molec/uc).

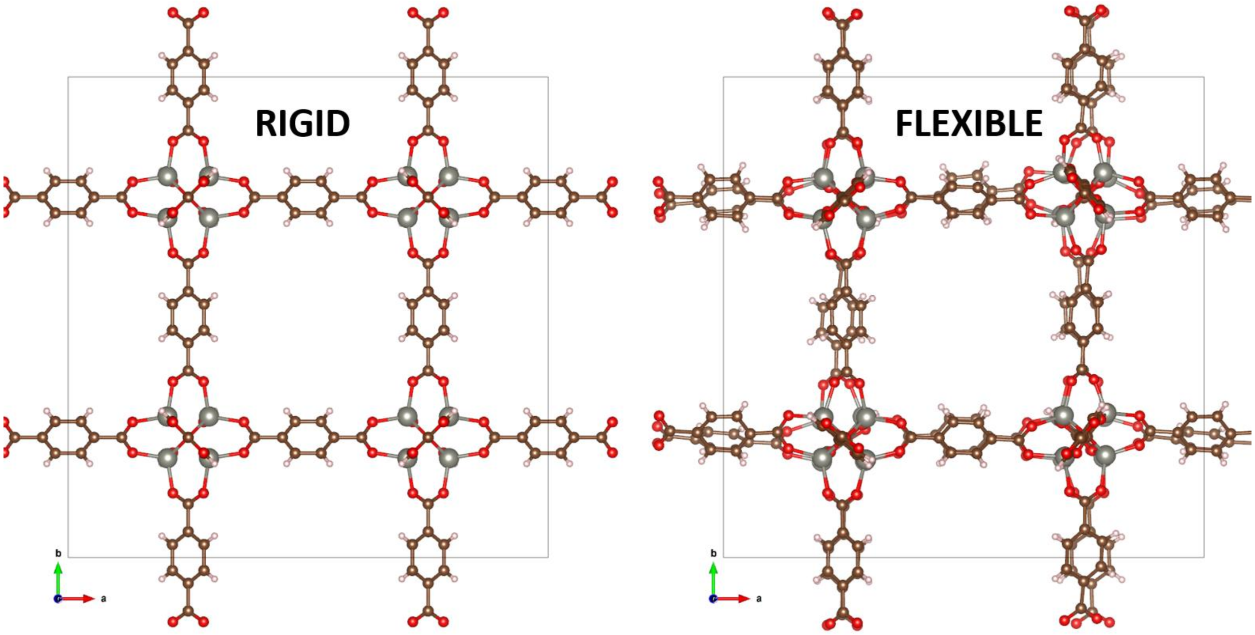

Figure S19. Framework of IRMOF-1 at the end of the MD + Widom simulations at 112 K at a loading of 237 cm^3/cm^3 (110 molec/uc) using rigid (left) and flexible (right) framework models.

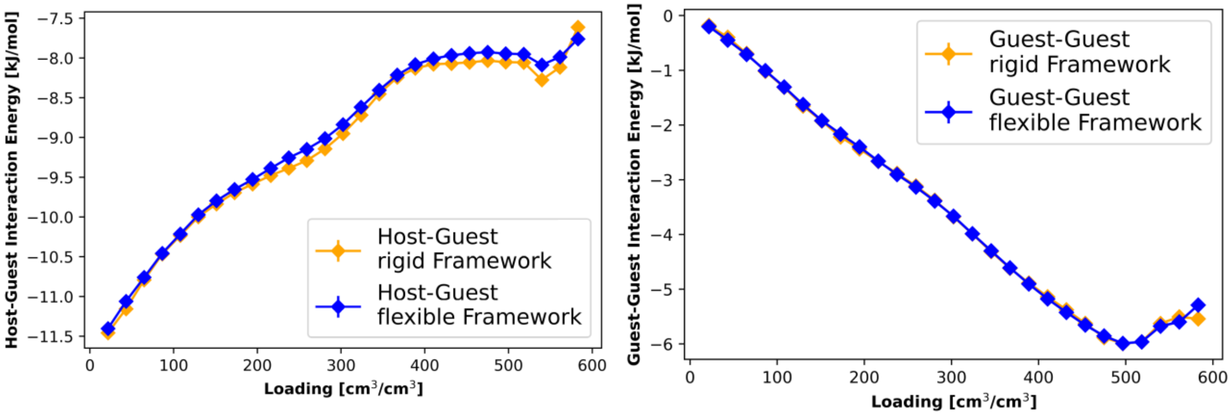

Figure S20. Host-guest and guest-guest interaction energies for methane in IRMOF-1 at 112 K (T_r = 0.587) simulated by MD + Widom insertions using the rigid (orange curve) and flexible (blue) framework.

Figure S21 shows the difference in host-guest energy per molecule between the rigid and flexible framework models. The figure also includes the loadings for the spinodals and binodals of the vdW loops and the pore-filling. We can see that as the loading increases beyond the low-density spinodal of the vdW loop (blue dashed vertical line), the difference in the host-guest interaction increases. The difference peaks after reaching the high-density spinodal, and then for loadings higher than the high-density binodal (red vertical line), between 280 cm^3/cm^3 (130 molec/uc) and 350 cm^3/cm^3 (162 molec/uc), the difference in host-guest interaction energy decreases. The isotherms in Figure S17 also show that the discrepancy in the two canonical isotherms is smaller in this regime. However, as the loading increases further, the system enters the pore-filling region and the difference in the host-guest interaction energy starts to increase again. We can see that for this region in the isotherms in Figure S17, the difference in the two isotherms also increases. This difference is related to the steric hindrance near the saturation and causes the saturation loading of the flexible simulation to be lower than the rigid one.

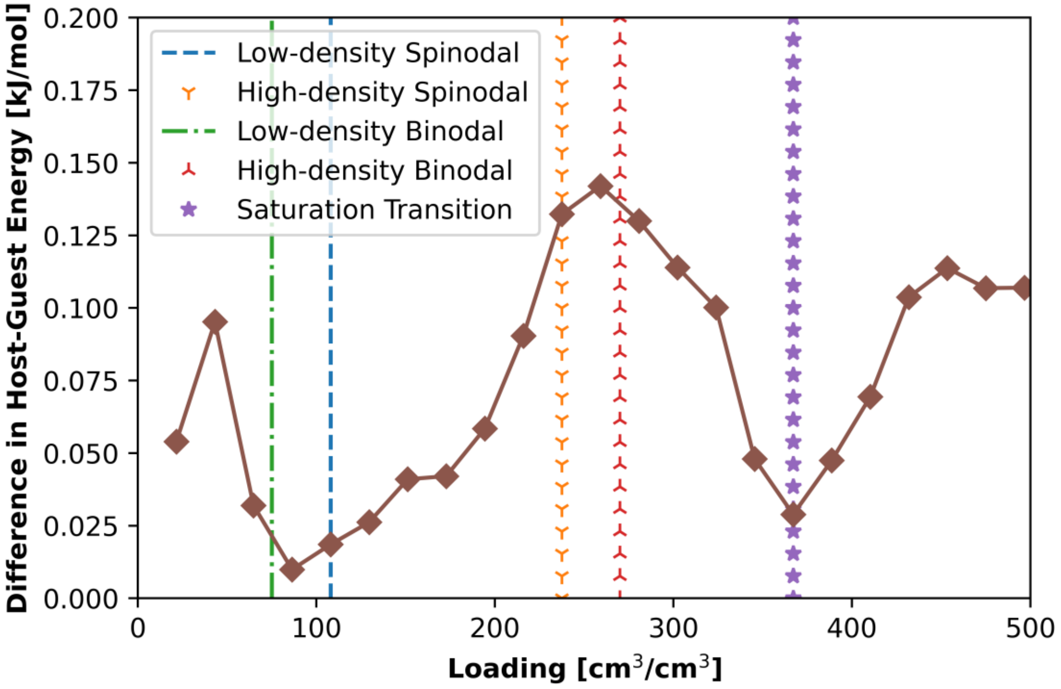

Figure S21. Difference in the host-guest interaction energy (flexible minus rigid). The brown curve shows the difference at various loadings. The loadings for the spinodals and binodals of the vdW loop are shown on the graph. The pore-filling is also shown in the figure using the purple stars.

Sample Input Files

Canonical Widom Insertion simulation:

```
SimulationType              MonteCarlo
NumberOfCycles              1000000
NumberOfEquilibrationCycles   20000
NumberOfInitializationCycles  20000
PrintEvery                  1000
RestartFile                 no
ContinueAfterCrash no
WriteBinaryRestartFileEvery 1000

RemoveAtomNumberCodeFromLabel yes
ChargeMethod None

Forcefield                  UFF
Cutoff                      12.8

Framework 0
FrameworkName 667
UnitCells 1 1 1
ExternalTemperature 95.28

Movies yes
WriteMoviesEvery 100000
LMPData yes

NumberOfGrids 1
GridTypes CH4_sp3
SpacingVDWGrid 0.1
```

UseTabularGrid yes

Component 0 MoleculeName methane

MoleculeDefinition TraPPE

TranslationProbability 1.0

ReinsertionProbability 1.0

WidomProbability 2.0

CreateNumberOfMolecules 10

Canonical Molecular Simulation + Widom Insertion

SimulationType MolecularDynamics

NumberOfCycles 1500000

NumberOfEquilibrationCycles 10000

NumberOfInitializationCycles 10000

PrintEvery 1000

RestartFile no

ContinueAfterCrash no

WriteBinaryRestartFileEvery 1000

#RemoveAtomNumberCodeFromLabel yes

ChargeMethod Ewald

Ensemble NVT

TimeStep 0.002

Forcefield Dubbeldam2007FlexibleIRMOF-1

Cutoff 12.8

Framework 0

```
FrameworkName IRMOF-1
UnitCells 1 1 1
ExternalTemperature 111.58

NumberOfWidomEach          200

FlexibleFramework yes
FrameworkDefinitions Dubbeldam2007FlexibleIRMOF-1

Movies yes
WriteMoviesEvery 30000

Component 0 MoleculeName           methane
          MoleculeDefinition       TraPPE
          TranslationProbability        1.0
          ReinsertionProbability        1.0
          WidomProbability        1.0
          CreateNumberOfMolecules  10
```

Supplementary References

(1) Dubbeldam, D.; Walton, K. S.; Ellis, D. E.; Snurr, R. Q. Exceptional Negative Thermal Expansion in Isoreticular Metal–Organic Frameworks. *Angewandte Chemie* **2007**, *119* (24), 4580–4583. https://doi.org/10.1002/ange.200700218.

(2) Friend, D. G.; Ely, J. F.; Ingham, H. Thermophysical Properties of Methane. *Journal of Physical and Chemical Reference Data* **1989**, *18* (2), 583–638. https://doi.org/10.1063/1.555828.

(3) Martin, M. G.; Siepmann, J. I. Transferable Potentials for Phase Equilibria. 1. United-Atom Description of n-Alkanes. *J. Phys. Chem. B* **1998**, *102* (14), 2569–2577. https://doi.org/10.1021/jp972543+.

(4) Jangkamolkulchai, A.; Luks, K. D. Partial Miscibility Behavior of the Methane + Ethane + N-Docosane and the Methane + Ethane + n-Tetradecylbenzene Ternary Mixtures. *J. Chem. Eng. Data* **1989**, *34* (1), 92–99. https://doi.org/10.1021/je00055a027.

(5) Shah, M. S.; Siepmann, J. I.; Tsapatsis, M. Transferable Potentials for Phase Equilibria. Improved United-Atom Description of Ethane and Ethylene. *AIChE Journal* **2017**, *63* (11), 5098–5110. https://doi.org/10.1002/aic.15816.

(6) Teja, A. S.; Lee, R. J.; Rosenthal, D.; Anselme, M. Correlation of the Critical Properties of Alkanes and Alkanols. *Fluid Phase Equilibria* **1990**, *56*, 153–169. https://doi.org/10.1016/0378-3812(90)85100-O.
