# Supplementary material for: Computational investigation of hysteresis and phase equilibria of n-alkanes in a metal-organic framework with both micropores and mesopores
Source: Commun Chem. 2023 May 8;6:90. doi: 10.1038/s42004-023-00889-3 (PMC10167368; doi:10.1038/s42004-023-00889-3)
Supplement: Supplementary file 3 — Description of Additional Supplementary Files [file 42004_2023_889_MOESM3_ESM.pdf]

Description of Additional Supplementary Files

File name: Supplementary Data 1 (Python-Code.zip)

Description: This zip file contains the Python scripts for processing the simulated canonical isotherm data and calculating the spinodal and binodal from the canonical isotherm.

File name: Supplementary Data 2 (NVT-Widom.zip)

Description: This zip file contains general input files for RASPA-2.0 to simulate canonical isotherms of adsorbates in MOFs.

File name: Supplementary Data 3 (IRMOF-1-MDWidom.zip)

Description: This zip file contains input files for RASPA-2.0 to simulate canonical isotherms of methane in flexible IRMOF-1 at 112 K.
