# Supplementary figures and images for: Computational investigation of hysteresis and phase equilibria of n-alkanes in a metal-organic framework with both micropores and mesopores
Source: Commun Chem. 2023 May 8;6:90. doi: 10.1038/s42004-023-00889-3 (PMC10167368; doi:10.1038/s42004-023-00889-3)

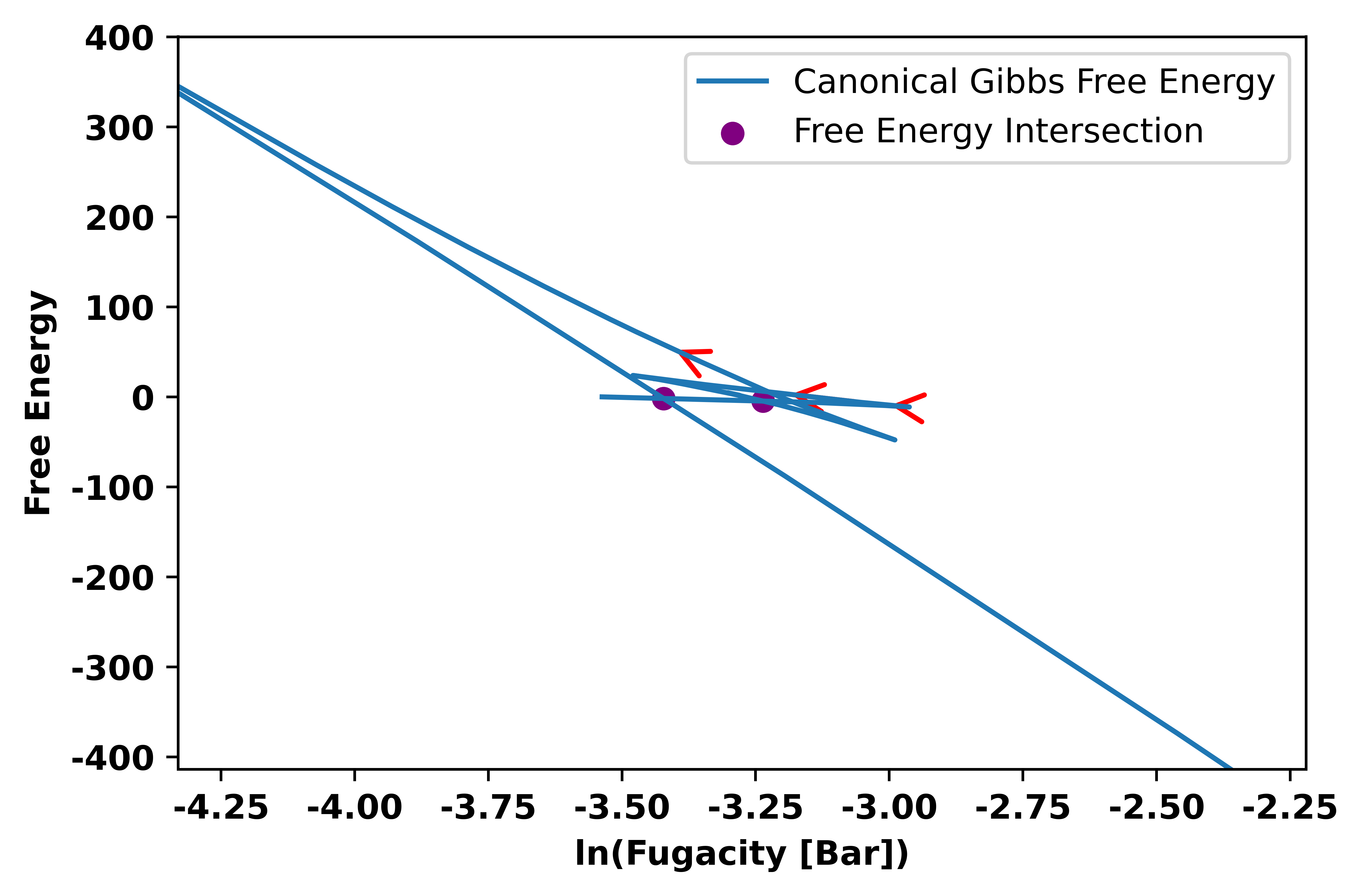

Supplement: Supplementary file 4 — Supplementary Data 1 [file 42004_2023_889_MOESM4_ESM.zip › Python-Code/0.5-Gibbs-real.png]

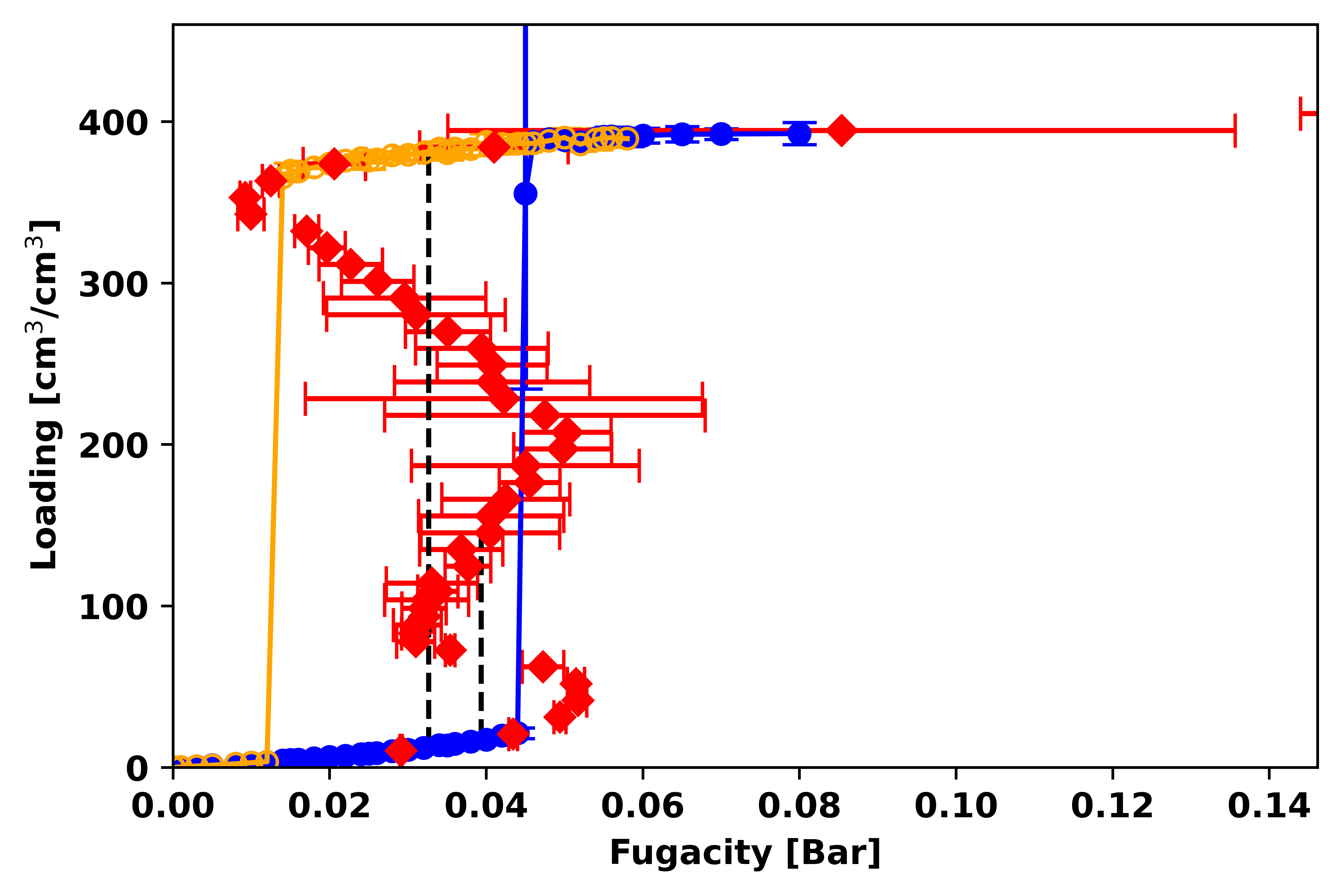

Supplement: Supplementary file 4 — Supplementary Data 1 [file 42004_2023_889_MOESM4_ESM.zip › Python-Code/0.5-MDW-Isotherm-Error.png]

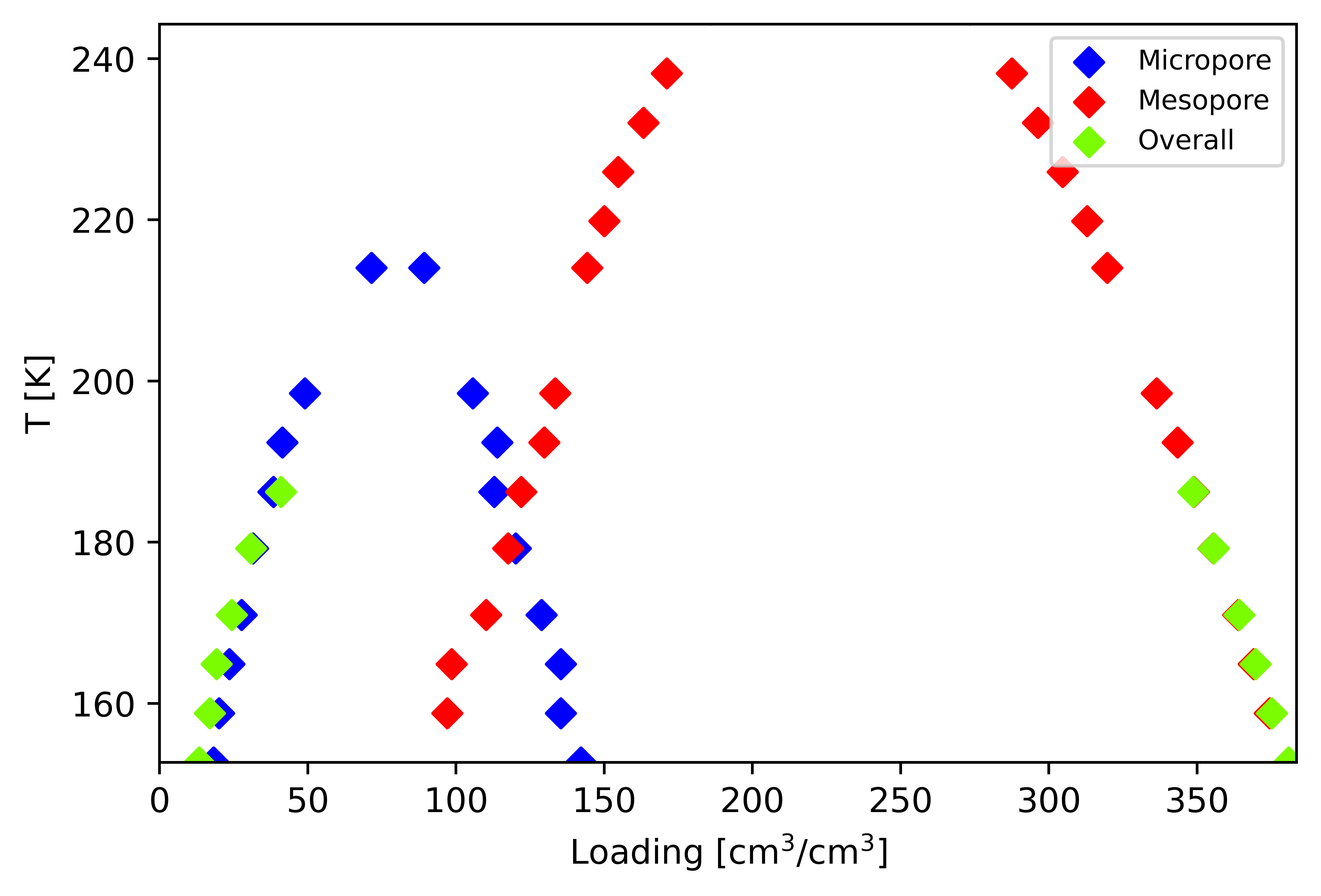

Supplement: Supplementary file 4 — Supplementary Data 1 [file 42004_2023_889_MOESM4_ESM.zip › Python-Code/VLE-Binodal.png]
